# Comparison of maxillomandibular asymmetries in adult patients presenting different sagittal jaw relationships

**DOI:** 10.1590/2177-6709.24.4.054-062.oar

**Published:** 2019

**Authors:** Guilherme Thiesen, Maria Perpétua Mota Freitas, Bruno Frazão Gribel, Ki Beom Kim

**Affiliations:** 1 Universidade do Sul de Santa Catarina, Departamento de Ortodontia (Florianópolis/SC, Brazil).; 2 Universidade Luterana do Brasil, Departamento de Ortodontia (Canoas/RS, Brazil).; 3 Private practice (Belo Horizonte/MG, Brazil).; 4 Saint Louis University, Department of Orthodontics (Saint Louis/MO, USA).

**Keywords:** Facial asymmetry, Malocclusions, 3-D imaging, Cone-beam computed tomography

## Abstract

**Objective:**

The present study aims at using cone beam computed tomography (CBCT) to analyze the maxillomandibular characteristics present in adults with mandibular asymmetries and different sagittal jaw relationships.

**Methods:**

360 patients were selected and divided into three groups (Class I, Class II, and Class III), with 120 individuals per group. The groups were then subdivided according to the intensity of lateral deviation of the gnathion point, into: 1) relative symmetry, 2) moderate asymmetry, and 3) severe asymmetry. Three planes of reference were established in the CBCT images and several measurements were taken to compare the bilateral skeletal differences between the intensities of asymmetry for the different sagittal jaw relationships.

**Results:**

When the groups were compared by the intensity of asymmetry, significant differences among patients with relative symmetry and moderate to severe asymmetry were found. This was especially noticed for severe asymmetry, suggesting that the deviation of the chin did not constitute the only morphological alteration for these patients, especially because a series of measurements showed significant bilateral differences. When comparing sagittal jaw relationships, the only significant finding was the vertical positioning of the gonion between Class II and III patients with severe asymmetry.

**Conclusions:**

When comparing the three sagittal jaw relationships with the same intensity of asymmetry, most maxillofacial aspects were quite similar. The only difference was found for patients with severe asymmetry, as the individuals with Class II showed greater bilateral difference in the vertical positioning of the gonion, when compared to patients with Class III.

## RESUMO

**Objetivo:**

o presente estudo teve como objetivo analisar, por meio de tomografia computadorizada de feixe cônico (TCFC), as características esqueléticas maxilomandibulares presentes em adultos com diferentes graus de assimetria mandibular e diferentes relações esqueléticas sagitais.

**Métodos:**

foram selecionados 360 pacientes, os quais foram divididos em três grupos, com 120 indivíduos cada (Classe I, Classe II e Classe III). Cada grupo foi, ainda, subdividido de acordo com a intensidade do desvio lateral do mento: 1) simetria relativa; 2) assimetria moderada; e 3) assimetria severa. Três planos de referência foram estabelecidos nas imagens de TCFC e várias medidas foram realizadas, sendo comparadas as diferenças esqueléticas bilaterais existentes entre os graus de assimetria em cada grupo, nas diferentes relações esqueléticas sagitais.

**Resultados:**

quando comparados os grupos pelos graus de assimetria, existiram diferenças significativas entre pacientes com simetria relativa e pacientes assimétricos, especialmente para a assimetria severa, sugerindo que o desvio do mento não constitui a única alteração morfológica para esses pacientes, especialmente porque uma série de medidas apresentou diferenças bilaterais significativas. Quando comparadas as relações sagitais entre si, não foram encontradas diferenças significativas, exceto para o posicionamento vertical do gônio entre as Classe II e III na assimetria severa.

**Conclusão:**

ao comparar a mesma intensidade de assimetria entre as diferentes relações esqueléticas sagitais, pouca diferença pôde ser observada. A única diferença encontrada foi em pacientes com assimetria severa, na qual os indivíduos com Classe II apresentaram maior diferença bilateral no posicionamento vertical do gônio, quando comparados àqueles com Classe III.

## INTRODUCTION

Lateral deviation of the chin is considered the most striking characteristic of facial asymmetry. This type of asymmetry has a prevalence generally reported to be between 11 and 37%^1^ ^-^ ^5^ in patients who seek orthodontic treatment. When present, this incongruence commonly presents as characteristically unfavorable to the patient from the esthetic and functional point of view, as well as a challenge for the clinician providing the treatment.

Such asymmetries may have specific pathological factors as etiologies, either congenital or acquired, as well as developmental alterations of undefined origin.^6^ ^-^ ^8^ Although there is no concrete response to explain the cause of these developmental asymmetries, some theories claim that accentuated mandibular growth could be more predisposed to complications of environmental and genetic factors. This aspect would make the asymmetry more evident.^8^ ^-^ ^10^

Some studies claim that, in terms of prevalence, mandibular asymmetries seem to be equally distributed among Class I, II, and III malocclusions.^11^ However, other studies have shown that such asymmetries would be more frequently related to patients with Class III,^5^ ^,^ ^12^ and less related to those with Class II.^2^

Despite these epidemiological differences, few studies in the literature have sought to compare the existing three-dimensional structural differences regarding asymmetry in the different sagittal jaw patterns. Some studies have compared Class III with Class I,^13^ ^-^ ^17^ Class II with Class I,^18^ or Class III with Class II,^19^ but none have compared the malocclusions fully. On this basis, the present article sought to analyze, using CBCT images, the maxillomandibular skeletal characteristics present in adults with different intensities of mandibular asymmetry and different sagittal jaw relationships.

## MATERIAL AND METHODS

Institutional ethical committee approval from *Universidade do Sul de Santa Catarina* was obtained prior to conducting the study (reference number: 1.591.220). All procedures were in accordance with the ethical standards of this committee on human experimentation (institutional and national) and with the Helsinki Declaration of 1975, as revised in 2008.

This cross-sectional study was nested within a previous epidemiological investigation that analyzed the prevalence and associations of mandibular asymmetries.^20^ CBCT images of 360 individuals were eligible, and power calculation for the statistical tests applied demonstrated that this sample size would suffice (β < 0.2, using α = 0.05).

All patients belonged to the database of a center for diagnostic services and dentistry planning. The tomographic images were obtained between 2011 and 2013.

The following inclusion criteria were adopted: individuals between the ages of 19 and 60 years with requested tomographic images when clinically justified, thus following the directives of the SedentexCT project and of the American Academy of Oral and Maxillofacial Radiology;^21^ ^,^ ^22^ and presence of all erupted permanent teeth (excluding the third molars). The exclusion criteria were: history of orthodontic treatment, fractures or surgery in the region of the face, degenerative disease in the temporomandibular joint, and craniofacial anomalies.

To conduct the exams, all images were obtained using the same type of tomographic equipment (iCAT^®^, Imaging Sciences International, Hatfield, PA), adjusted to the following specifications: extended field of acquisition (16 x 22cm or 17 x 23cm), 120KvP, 3-8mA and voxel pattern of 0.4mm^3^.

The patients were seated so that the head was positioned with the Frankfort plane parallel to the ground, the median sagittal plane perpendicular to the ground, and were instructed to close the mouth to maximum intercuspation and to let the lips relaxed.

The CBCT images were exported in DICOM (Digital Imaging and Communication in Medicine) format, using the iCAT Vision^®^ software. The DICOM files were imported into the SimPlant Ortho Pro^®^ 2.0 (Materialise Dental, Leuven, Belgium) software and the anatomical points were located according to the multiplanar reconstruction slices, using a measurement scale of 0.01mm and 0.01^o^.

The total sample was divided into three groups with 120 individuals each, according to the sagittal jaw patterns for Class I (ANB angle from 0 to 4.5^o^), Class II (ANB > 4.5^o^) and Class III (ANB < 0^o^), as proposed by Tweed.^23^ Each group was subdivided into three additional categories with 40 individuals each, according to the intensity of chin laterality. The lateral deviation of the gnathion point was the criterion established to determine mandibular asymmetry, since this deviation greatly influences the perception of an asymmetrical face.^2^ Patients with displacement of 2mm or less were defined as exhibiting relative symmetry. Patients whose gnathion was displaced by more than 2 mm and up to 4 mm were defined as exhibiting moderate asymmetry. Patients with gnathion displacement from the midsagittal plane greater than 4 mm were defined as exhibiting severe asymmetry. These parameters were adopted according to data suggested in other studies.^9^ ^,^ ^24^ ^-^ ^27^

**Figure 1:**
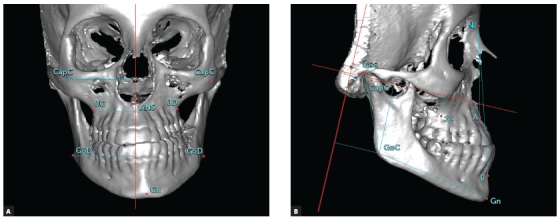
Measurements made: A) Gn-MSP, ANS-MSP, Go-MSP, J-MSP, Cap-MSP; B) ANB angle, Go-Coronal, Cap-Coronal, GoGn, CoGo, Go-Camper and J-Camper. For the bilateral points, measurements were obtained both on the contralateral side (C) and on the deviated side (D), as well as the difference between them (/dif).

Table 1 describes the landmarks and reference planes used in the present study. Three reference planes were established in the CBCT images and the mandibular and maxillary measurements were made and grouped in the transverse, sagittal, and vertical planes. The methodology used in the present study for determining the midsagittal plane was previously validated by the study of Damstra el al.^28^ These measurements are described in Table 2 and illustrated in Figure 1.

**Table 1:** Landmarks and reference planes used in the study.

Landmark/Plane	Abbreviation	Definition
Anatomic porion	Po	Most superior point of the external acoustic meatus
Orbitale	Or	Most inferior point of the infraorbital margin
Anterior nasal spine	ANS	Point located at the tip of the anterior nasal spine
Basion	Ba	Middle point on the anterior rim of the occipital foramen
Sella	S	Point in the center of the sella turcica
Nasion	N	Most anterior and median point of the frontonasal suture
Subspinale	A	Point located at the largest concavity of the anterior portion of the maxilla
Supramentale	B	Point located at the largest concavity of the anterior portion of the mental symphysis
Gnathion	Gn	Most anterior inferior point of the contour of the bony menton
Jugale	J	Point in the intersection of the contour of the maxillary tuberosity with the zygomatic pillar
Capitulare	Cap	Point in the center of the head (condyle) of the mandible
Gonion	Go	Most inferior and posterior point on the contour of the gonial angle
Condylion	Co	Most superior and posterior point of the mandibular condyle
Frankfort Plane	Frankfort	Plane passing through the right and left anatomic porion points and the left orbitale point (PoR, PoL - OrL)
Midsagittal Plane	MSP	Plane that refers to the junction of nasion and basion points, perpendicular to the Frankfort plane. Used to evaluate changes in the transversal direction
Coronal Plane	Coronal	Plane that passes through the points right and left anatomic porion, perpendicular to the Frankfort plane. Used to evaluate changes in the sagittal direction
Camper Plane	Camper	Plane that passes through the points right and left anatomic porion and the anterior nasal spine (ANS). Used to evaluate changes in the vertical direction

**Table 2:** Measurements performed to evaluate bilateral differences of mandibular and maxillary components.

	Variable	Measurement	Definition
Transverse	Gn-MSP	Distance from the gnathion to the midsagittal plane	Mandibular asymmetry (lateral deviation of the menton)
	ANS-MSP	Distance from the anterior nasal spine to the midsagittal plane	Maxillary asymmetry (lateral deviation of the anterior maxilla)
	Go-MSP	Distance from gonion to midsagittal plane, measured on contralateral and deviated sides	Transverse positioning of the gonion
	J-MSP	Distance from jugale point to midsagittal plane, measured on contralateral and deviated sides	Transverse positioning of the jugale (maxilla)
	Cap-MSP	Distance from capitulare to midsagittal plane, measured on contralateral and deviated sides	Transverse positioning of the head of the condyle
Sagital	ANB angle	Angle formed by the intersection of lines NA and NB	Sagittal jaw relationship
	Go-Coronal	Distance from gonion to coronal plane, measured on contralateral and deviated sides	Sagittal positioning of the gonion
	Cap-Coronal	Distance from capitulare to coronal plane, measured on contralateral and deviated sides	Sagittal positioning of the head of the condyle
	GoGn	Distance from gonion to gnathion, measured on contralateral and deviated sides	Length of the mandibular body
Vertical	CoGo	Distance from condylion to gonion, measured on contralateral and deviated sides	Height of the mandibular ramus
	Go-Camper	Distance from gonion to Camper plane, measured on contralateral and deviated sides	Vertical positioning of the gonion
	J-Camper	Distance from jugale point to Camper plane, measured on contralateral and deviated sides	Vertical positioning of the jugale
Transverse	Go-MSP/dif	Difference in the distance from gonion to midsagittal plane, measured on contralateral and deviated side	Bilateral difference of the position of the gonion point, in the transverse plane
	J-MSP/dif	Difference in the distance from the jugale point to midsagittal plane, measured on contralateral and deviated side	Bilateral difference of the position of the jugale point, in the transverse plane
	Cap-MSP/dif	Difference in the distance from capitulare to midsagittal plane, measured on contralateral and deviated side	Bilateral difference of the position of the head of the condyle, in the transverse plane
Sagital	Go-Coronal/dif	Difference in the distance from gonion to coronal plane, between contralateral and deviated sides	Bilateral difference of the position of the gonion point, in the sagittal plane
	Cap-Coronal/dif	Difference in the distance from capitulare to coronal plane, between contralateral and deviated sides	Bilateral difference of the position of the head of the condyle, in the sagittal plane
	GoGn/dif	Difference in the distance from gonion to gnathion, between contralateral and deviated sides	Bilateral difference of the lengths of mandibular bodies
Vertical	CoGo/dif	Difference in the distance from condylion to gonion, between contralateral and deviated sides	Bilateral difference of the heights of mandibular rami
	Go-Camper/dif	Difference in the distance from gonion to Camper plane, between contralateral and deviated sides	Bilateral difference of the position of the gonion point, in the vertical plane
	J-Camper/dif	Difference in the distance from the jugale point to Camper plane, between contralateral and deviated sides	Bilateral difference of the position of the jugale point, in the vertical plane

/dif = difference: value obtained in the contralateral side deducted from the deviated side.

The deviation of the gnathion to the midsagittal plane was considered in absolute values, independent of the side of the deviation. To determine the asymmetry between the measurements taken in bilateral cephalometric points, the difference (/dif) was analyzed between the contralateral side and the side of mandibular deviation.

To calculate the error of the method, 10% of the sample was evaluated at two separate times by the same evaluator, at a two-week interval. The IntraClass Correlation Coefficient (ICC) was used, and a value >0.80 was obtained for all measurements evaluated. 

Statistical analyses were conducted using the SPSS^®^20.0 (IBM, Chicago, IL, USA) software. The Shapiro-Wilk test was applied, showing normal distribution of the values obtained in bilateral measurements, and abnormal distribution of the values obtained in midpoints measurements. To verify possible differences among Classes I, II, and III patients in relation to the different intensities of asymmetry, the Analysis of Variance (complemented by the Tukey test) was conducted when the data showed normality, and the Kruskal-Wallis test was used when the normality criterion was not satisfied (complemented by the Mann-Whitney test with Bonferroni correction, to identify the differences). A 5% significance level was considered.

## RESULTS

Table 3 describes the distribution of the sample for frequency according to sex, in addition to the means, standard deviations, and amplitudes by age, ANB angle, and deviation from the gnathion point (in absolute values) for each sagittal jaw relationship evaluated.

**Table 3:** Characteristics of the sample according to sex, age, ANB Angle and lateral deviation from the gnathion.

	Class I (n=120)	Class II (n=120)	Class III (n=120)	Total sample (n=360)
Sex				
Male: n (%)	41 (34.2%)	37 (30.8%)	52 (43.3%)	130 (36.1%)
Female: n (%)	79 (65.8%)	83 (69.2%)	68 (56.7%)	230 (63.9%)
Age				
mean ± SD	30.58 ± 9.46;	30.72 ± 10.26;	26.54 ± 8.57;	29.28 ± 9.62;
range (min/max)	(19 / 57)	(19 / 54)	(19 / 56)	(19 / 57)
ANB				
mean ± SD	2.42 ± 1.16;	6.19 ± 1.42;	-2.92 ± 2.43;	1.89 ± 4.13;
range (min/max)	(0.06 / 4.49)	(4.52 / 12.26)	(-0.03 / -12.16)	(12.26 / -12.16)
Gn to MSP				
mean ± SD	3.39 ± 2.74;	3.21 ± 2.75;	3.38 ± 2.68;	3.33 ± 2.71;
range (min/max)	(0.04 / 11.85)	(0.01 / 21.49)	(0.10 / 12.41)	(0.01 / 21.49)

Table 4 shows the variables used in the analysis of the differences obtained between the contralateral side and the deviated side for maxillary and mandibular skeletal measurements, in each group of sagittal jaw relationships. Comparing the patients with relative symmetry, moderate asymmetry, and severe asymmetry, it was found that the Gn-MSP, Go-MSP/dif and CoGo/dif values differed significantly among the intensities of asymmetry for all groups.

**Table 4:** Comparison of the values obtained between the three intensities of mandibular asymmetry, in each sagittal jaw relationship.

		Class I				Class II				Class III			
	Variable	Relative Symmetry	Moderate Asymmetry	Severe Asymmetry	p	Relative Symmetry	Moderate Asymmetry	Severe Asymmetry	p	Relative Symmetry	Moderate Asymmetry	Severe Asymmetry	p
		(mean ± SD)	(mean ± SD)	(mean ± SD)		(mean ± SD)	(mean ± SD)	(mean ± SD)		(mean ± SD)	(mean ± SD)	(mean ± SD)	
Transverse Plane	Gn-MSP	0.80 ± 0.51 A	2.78 ± 0.60 B	6.60 ± 2.11 C	<0.001§	0.88 ± 0.63 A	2.72 ± 0.63 B	6.02 ± 2.88 C	<0.001§	0.90 ± 0.52 A	2.82 ± 0.52 B	6.43 ± 2.31 C	<0.001§
	ANS-MSP	1.03 ± 0.91	1.07 ± 0.78	1.49 ± 1.10	0.189§	0.66 ± 0.60 A	1.60 ± 1.16 B	1.65 ± 1.18 B	<0.001§	0.88 ± 0.68	0.97 ± 0.37	1.33 ± 0.96	0.352§
	Go-MSP/dif	-0.54 ± 2.41 A	-2.19 ± 2.21 B	-4.04 ± 3.67 C	<0.001†	-0.69 ± 2.26 A	-2.55 ± 2.36 B	-4.34 ± 4.24 C	<0.001†	-0.52 ± 2.85 A	-1.47 ± 2.10 A	-3.73 ± 3.97 B	<0.001†
	J-MSP/dif	-0.12 ± 1.90 A	-0.81 ± 1.48 AB	-1.57 ± 2.22 B	0.004†	-0.87 ± 1.55	-0.88 ± 1.62	-1.73 ± 2.64	0.092†	-0.38 ± 1.36	-0.61 ± 1.37	-0.96 ± 1.94	0.254†
	Cap-MSP/dif	-0.24 ± 1.59	0.14 ± 2.83	0.06 ± 2.83	0.761†	-0.32 ± 1.89	-0.32 ± 2.01	-0.51 ± 4.36	0.951†	-0.24 ± 2.03	-0.06 ± 2.31	0.09 ± 2.67	0.813†
	Go-Coronal/dif	0.41 ± 2.42 A	0.45 ± 2.64 A	2.24 ± 2.89 B	0.003†	-0.17 ± 2.66	-0.02 ± 2.65	1.27 ± 4.34	0.102†	0.40 ± 2.31 A	0.83 ± 2.13 A	2.63 ± 3.08 B	<0.001†
Sagittal Plane	Cap-Coronal/dif	0.09 ± 1.06	0.23 ± 1.27	0.31 ± 1.11	0.682†	-0.01 ± 0.96	-0.07 ± 1.40	-0.22 ± 2.73	0.721†	0.10 ± 1.12 A	0.12 ± 1.06 A	1.03 ± 2.21 B	0.012†
	GoGn/dif	0.30 ± 2.04 A	0.42 ± 1.76 A	1.67 ± 2.22 B	0.005†	0.48 ± 1.94	0.73 ± 1.38	1.06 ± 3.91	0.616†	0.27 ± 2.17 A	0.89 ± 1.64 AB	2.04 ± 2.88 B	0.003†
	CoGo/dif	0.19 ± 2.37 A	1.29 ± 2.39 A	3.82 ± 3.85 B	<0.001†	-0.09 ± 2.79 A	1.07 ± 2.12 A	4.97 ± 6.14 B	<0.001†	-0.24 ± 2.49 A	0.50 ± 2.54 A	3.26 ± 5.16 B	<0.001†
Vertical Plane	Go-Camper/dif	0.01 ± 2.39 A	1.12 ± 2.92 A	2.91 ± 3.92 B	<0.001†	-0.01 ± 2.36 A	1.32 ± 2.03 A	3.88 ± 3.78 B	<0.001†	-0.03 ± 2.74	0.26 ± 2.79	1.58 ± 3.93	0.056†
	J-Camper/dif	0.01 ± 1.37 A	1.03 ± 1.08 B	1.09 ± 1.37 B	<0.001†	0.43 ± 1.04 A	0.48 ± 1.77 B	1.56 ± 1.98 B	0.003†	0.12 ± 1.57	1.01 ± 1.61	0.71 ± 1.81	0.058†

/dif = difference: value obtained in the contralateral side deducted from the deviated side. † Analysis of variance (ANOVA) complemented by a multiple comparison Tukey test. § Kruskal-Wallis test, followed by the Mann-Whitney test to identify intergroup differences. For each intensity of mandibular asymmetry, averages followed by distinct letters differ significantly, with a significance level of 5%.

The ANS-MSP variable showed a significant difference between symmetrical and asymmetrical only for Class II patients. There was a statistically significant difference for J-MSP/dif among Class I patients with mandibular symmetry and severe asymmetry. The Go-Coronal/dif variable showed significant differences for Class I and Class III patients when comparing patients with severe asymmetry and the others. There was a statistically significant difference for Cap-Coronal/dif between patients with severe asymmetry and the others only for Class III. The GoGn/dif variable showed significant differences between patients with severe asymmetry and the other patients in Class I, while in Class III the differences were only between severe asymmetry and relative symmetry. Significant differences were found for Go-Camper/dif among patients with severe asymmetry and the others in Classes I and II. Significant differences were found for J-Camper/dif between the symmetrical and asymmetrical Class I and II patients (Table 4).

When comparing Class I, II, and III patients (Table 5), in relation to the different intensities of asymmetry, it was found that there were no differences in the variables analyzed for relative symmetry and moderate asymmetry. Among the groups, the Go-Camper/dif measurement only differed in severe asymmetry, specifically between Class II and Class III patients. The Go-Camper/dif measurement in Class II patients with severe asymmetry was statically greater than this same measurement for Class III patients with severe asymmetry, suggesting a greater difference in the vertical position of the gonion point between the contralateral and the deviated sides.

**Table 5:** Comparison of the values obtained between each sagittal jaw relationship, comparing the intensities of mandibular asymmetry independently.

		Relative symmetry				Moderate asymmetry				Severe asymmetry			
	Variable	Class I (mean ± SD)	Class II (mean ± SD)	Class III (mean ± SD)	p	Class I (mean ± SD)	Class II (mean ± SD)	Class III (mean ± SD)	p	Class I (mean ± SD)	Class II (mean ± SD)	Class III (mean ± SD)	p
Transverse Plane	Gn-MSP	0.80 ± 0.51	0.88 ± 0.63	0.90 ± 0.52	0.703§	2.78 ± 0.60	2.72 ± 0.63	2.82 ± 0.52	0.783§	6.60 ± 2.11	6.02 ± 2.88	6.43 ± 2.31	0.565§
	ANS-MSP	1.03 ± 0.91	0.66 ± 0.60	0.88 ± 0.68	0.192§	1.07 ± 0.78	1.60 ± 1.16	0.97 ± 0.37	0.104§	1.49 ± 1.10	1.65 ± 1.18	1.33 ± 0.96	0.723§
	Go-MSP/dif	-0.54 ± 2.41	-0.69 ± 2.26	-0.52 ± 2.85	0.947†	-2.19 ± 2.21	-2.55 ± 2.36	-1.47 ± 2.10	0.094†	-4.04 ± 3.67	-4.34 ± 4.24	-3.73 ± 3.97	0.788†
	J-MSP/dif	-0.12 ± 1.90	-0.87 ± 1.55	-0.38 ± 1.36	0.112†	-0.81 ± 1.48	-0.88 ± 1.62	-0.61 ± 1.37	0.712†	-1.57 ± 2.22	-1.73 ± 2.64	-0.96 ± 1.94	0.289†
	Cap-MSP/dif	-0.24 ± 1.59	-0.32 ± 1.89	-0.24 ± 2.03	0.974†	0.14 ± 2.83	-0.32 ± 2.01	-0.06 ± 2.31	0.682†	0.06 ± 2.83	-0.51 ± 4.36	0.09 ± 2.67	0.666†
	Go-Coronal/dif	0.41 ± 2.42	-0.17 ± 2.66	0.40 ± 2.31	0.481†	0.45 ± 2.64	-0.02 ± 2.65	0.83 ± 2.13	0.311†	2.24 ± 2.89	1.27 ± 4.34	2.63 ± 3.08	0.207†
Sagittal Plane	Cap-Coronal/dif	0.09 ± 1.06	-0.01 ± 0.96	0.10 ± 1.12	0.862†	0.23 ± 1.27	-0.07 ± 1.40	0.12 ± 1.06	0.539†	0.31 ± 1.11	-0.22 ± 2.73	1.03 ± 2.21	0.059†
	GoGn/dif	0.30 ± 2.04	0.48 ± 1.94	0.27 ± 2.17	0.889†	0.42 ± 1.76	0.73 ± 1.38	0.89 ± 1.64	0.414†	1.67 ± 2.22	1.06 ± 3.91	2.04 ± 2.88	0.364†
	CoGo/dif	0.19 ± 2.37	-0.09 ± 2.79	-0.24 ± 2.49	0.732†	1.29 ± 2.39	1.07 ± 2.12	0.50 ± 2.54	0.307†	3.82 ± 3.85	4.97 ± 6.14	3.26 ± 5.16	0.322†
Vertical Plane	Go-Camper/dif	0.01 ± 2.39	-0.01 ± 2.36	-0.03 ± 2.74	0.997†	1.12 ± 2.92	1.32 ± 2.03	0.26 ± 2.79	0.075†	2.91 ± 3.92 AB	3.88 ± 3.78 A	1.58 ± 3.93 B	0.041†
	J-Camper/dif	0.01 ± 1.37	0.43 ± 1.04	0.12 ± 1.57	0.321†	1.03 ± 1.08	0.48 ± 1.77	1.01 ± 1.61	0.190†	1.09 ± 1.37	1.56 ± 1.98	0.71 ± 1.81	0.097†

/dif = difference: value obtained in the contralateral side deducted from the deviated side. † Analysis of variance (ANOVA) complemented by a multiple comparison Tukey test. § Kruskal-Wallis test, followed by the Mann-Whitney test to identify intergroup differences. For each sagittal jaw relationship, averages followed by distinct letters differ significantly, with a significance level of 5%.

## DISCUSSION

The term fluctuating asymmetry refers to the small, random variations in characteristics presumably having bilateral symmetry and is broadly used as a measure of instability in the development of plants and animals.^8^ ^,^ ^29^ As every human face has some degree of asymmetry, we may consider that only the moderate and severe asymmetries may require orthodontic treatment, including orthognathic surgery in the most serious cases.^6^ Therefore, knowledge of the factors determining facial asymmetry is essential for the orthodontist to properly diagnose the patient and establish the best treatment plan.

The present study revealed that there were marked differences between the maxillary and mandibular components that affect the different intensities of mandibular asymmetry (Table 4). The deviation of the chin is not the only morphological alteration for asymmetrical patients, since many of the variables analyzed showed significant differences. This fact is extremely important for the diagnosis and elaboration of a treatment plan for these patients, especially in cases involving orthognathic surgery. The measurements that evaluate the positioning of the gonion point are worth mentioning, as reported by other authors.^14^ ^,^ ^18^ ^,^ ^26^ These striking alterations in the three-dimensional positioning of the gonion point in asymmetrical patients may be related to unbalanced musculature in such individuals.^30^

In the present study, statistically significant (*p*< 0.05, Table 4) differences were found among the intensities of asymmetry in each group (Classes I, II and III) for the variables used to evaluate the lateral deviation of the gnathion, the bilateral difference in the lateral positioning of the gonion, and ramus height.

However, the main objective of the present study was to compare different sagittal jaw relationships. The present results showed that, when comparing the same intensities of asymmetry in the different sagittal jaw relationships, little differences were found (Table 5).

There was a statistically significant difference only between Class II and III patients with severe asymmetry for the variable used to analyze the bilateral differences in the vertical positioning of the gonion point. The results of the present study suggest that Class II patients with severe asymmetry presented a greater bilateral difference in the vertical positioning of the gonion, compared to Class III patients with severe asymmetry. This indicates that in Class II patients, the gonion on the contralateral side is commonly positioned below the gonion of the mandibular deviation side, and this difference is statistically greater than that of Class III patients (although the lower positioning of the gonion on the contralateral side also exists in Class III patients). This lower positioning of the gonion on the contralateral side was commonly seen in most patients with severe asymmetry, although individual variations were found. This is illustrated by the high standard deviation shown for this variable.

Similar to the present study, the study by Kim et al^19^ compared Classes II and III asymmetrical patients, and found that the only difference was a greater bilateral difference in the height of the mandibular ramus in Class II patients.

Sievers et al^18^ compared the index of asymmetry of cephalometric points in Classes I and II patients using CBCT and found no difference among them.

Studies that have made three-dimensional comparisons of the structural differences between Class I and Class III^13-17^ patients used Class I as the control, and the individuals of the control group were considered to have craniofacial symmetry. The differences found in these studies were statistically significant among the individuals for many of the variables analyzed, in the same way as in the present study when symmetrical and asymmetrical patients were compared.

The findings of the present study have highlighted the fact that the maxillomandibular components that show bilateral incongruence are commonly the same for patients with skeletal Classes I, II, and III who present the same degree of mandibular asymmetry. This suggests that asymmetries, when present, behave in a similar manner regardless of sagittal jaw pattern. The present study is also clinically relevant since it allows professionals to evaluate the morphologic components related to different intensities of chin deviation and correctly diagnose and define treatment plan for those patients. However, it is worth highlighting that some of the measures evaluated in the present study had considerable standard deviations. This indicates that the individual variation should be considered when evaluating the determinant morphological characteristics of craniofacial asymmetry.

## CONCLUSIONS

The three sagittal jaw relationships were compared for the maxillomandibular characteristics associated with different intensities of mandibular asymmetry. The main findings were as follows:

1. The deviation of the chin is not the only skeletal alteration for asymmetrical patients, since many of the analyzed variables showed significant differences when the intensities of the deviation were compared.
2. Few differences were found among the various sagittal jaw relationships. Class II patients showed a greater bilateral difference in the vertical positioning of the gonion, when compared to Class III patients, only in the severe asymmetry group.
